# Lipid Body Organelles within the Parasite *Trypanosoma cruzi*: A Role for Intracellular Arachidonic Acid Metabolism

**DOI:** 10.1371/journal.pone.0160433

**Published:** 2016-08-04

**Authors:** Daniel A. M. Toledo, Natália R. Roque, Lívia Teixeira, Erix A. Milán-Garcés, Alan B. Carneiro, Mariana R. Almeida, Gustavo F. S. Andrade, Jefferson S. Martins, Roberto R. Pinho, Célio G. Freire-de-Lima, Patrícia T. Bozza, Heloisa D’Avila, Rossana C. N. Melo

**Affiliations:** 1 Laboratory of Cellular Biology, Department of Biology, Federal University of Juiz de Fora (UFJF), Juiz de Fora, MG, Brazil; 2 Laboratory of Immunopharmacology, Oswaldo Cruz Institute, FIOCRUZ, Rio de Janeiro, Brazil; 3 Laboratory of Plasmonic Nanostructures, Molecular Spectroscopy and Structure Group, Department of Chemistry, Federal University of Juiz de Fora (UFJF), Juiz de Fora, MG, Brazil; 4 Department of Physics, Federal University of Juiz de Fora (UFJF), Juiz de Fora, MG, Brazil; 5 Laboratory of Immunomodulation, Institute of Biophysics Carlos Chagas Filho, Federal University of Rio de Janeiro (UFRJ), Rio de Janeiro, Brazil; Instituto Butantan, BRAZIL

## Abstract

Most eukaryotic cells contain varying amounts of cytosolic lipidic inclusions termed lipid bodies (LBs) or lipid droplets (LDs). In mammalian cells, such as macrophages, these lipid-rich organelles are formed in response to host-pathogen interaction during infectious diseases and are sites for biosynthesis of arachidonic acid (AA)-derived inflammatory mediators (eicosanoids). Less clear are the functions of LBs in pathogenic lower eukaryotes. In this study, we demonstrated that LBs, visualized by light microscopy with different probes and transmission electron microscopy (TEM), are produced in trypomastigote forms of the parasite *Trypanosoma cruzi*, the causal agent of Chagas’ disease, after both host interaction and exogenous AA stimulation. Quantitative TEM revealed that LBs from amastigotes, the intracellular forms of the parasite, growing in vivo have increased size and electron-density compared to LBs from amastigotes living in vitro. AA-stimulated trypomastigotes released high amounts of prostaglandin E_2_ (PGE_2_) and showed PGE_2_ synthase expression. Raman spectroscopy demonstrated increased unsaturated lipid content and AA incorporation in stimulated parasites. Moreover, both Raman and MALDI mass spectroscopy revealed increased AA content in LBs purified from AA-stimulated parasites compared to LBs from unstimulated group. By using a specific technique for eicosanoid detection, we immunolocalized PGE_2_ within LBs from AA-stimulated trypomastigotes. Altogether, our findings demonstrate that LBs from the parasite *Trypanosoma cruzi* are not just lipid storage inclusions but dynamic organelles, able to respond to host interaction and inflammatory events and involved in the AA metabolism. Acting as sources of PGE_2_, a potent immunomodulatory lipid mediator that inhibits many aspects of innate and adaptive immunity, newly-formed parasite LBs may be implicated with the pathogen survival in its host.

## Introduction

The dynamic nature of lipid bodies (LBs), also known as lipid droplets has lead to their recognition as highly active organelles within most cell types involved in different biological functions and containing not only lipids but also many proteins (reviewed in [1–3]). During the last decades, the structure and function of these lipid-rich organelles, which are surrounded by a single layer of phospholipids, have been consistently investigated in mammalian cells and attracted great attention due to their association with human diseases (reviewed in [1–4]).

LB biogenesis is a well-documented process that happens *in vivo* within many types of mammalian cells during inflammatory reactions of varied causes, including infectious diseases with different pathogens such as bacteria, parasites and virus. Host-pathogen interaction leads to increased formation of LBs within cells from the immune system mainly macrophages. In these cells, LBs serve as intracellular sites for metabolic transformation of arachidonic acid (AA) into biologically active inflammatory mediators (eicosanoid derivatives) (reviewed in [5–7]). Thus, LBs in mammalian cells are remarkably linked to inflammatory responses and are considered structural markers of inflammation [5, 6, 8].

In recent years, there has been a renaissance of interest and recognition in the structure, composition and function of lipid-rich organelles formed within pathogenic prokaryotes and lower eukaryotes. In the past, pathogen-derived LBs were mostly considered as lipid deposits with slow rates of turnover (reviewed in [1]). However, evidence begins to accumulate that cytosolic LBs of intracellular bacteria and parasitic protozoa have more dynamic roles. These pathogens are able to usurp host lipids or to encode their own lipid biosynthesis machinery, thus allowing formation of LBs independently of their host (reviewed in [9, 10]). For example, the infection of erythrocytes with the malaria parasite *Plasmodium falciparum* induces LBs formation within the parasite [11]. These newly synthesized LBs accumulate in the food parasite vacuole and are involved in the detoxification of *heme*, enabling parasite persistence [11]. During the infection with *Toxoplasma gondii* [12] or *Mycobacterium tuberculosis* [13], host-derived lipids are imported and used for the synthesis of cholesteryl esters or triglycerides that are deposited in pathogen LBs. More recently, it was demonstrated that LBs numbers as well as the expression of prostaglandin F_2_ alpha (PGF_2_α) synthase (PGFS) increases during the development of *Leishmania infantum chagasi* to a virulent metacyclic stage [14].

Here, we studied the formation of LBs within the parasite *Trypanosoma cruzi* and the ability of parasite-derived LBs to produce eicosanoid in response to exogenous AA. *T*. *cruzi* is an obligate intracellular parasite capable of infecting different types of nucleated cells of humans and warm-blooded animals, and responsible for Chagas’ disease, which remains a major problem with a great impact on public health in Latin America [15].

We demonstrate, for the first time, that *T*. *cruzi* LBs are formed and respond to both host interaction and AA-stimulation, are able to incorporate AA and can be sources of prostaglandin E_2_ (PGE_2_), a potent immunomodulatory lipid mediator known to inhibit many aspects of innate and adaptive immunity [16–18]. Our results raise the possibility that pathways of AA metabolism of potential pathophysiologic significance may exist within human-living *T*. *cruzi*.

## Materials and Methods

### Animals and infection

Peritoneal macrophages from uninfected C57BL/6 mice were plated in RPMI-1640 medium plus 10% fetal bovine serum, 1% streptomicin/penicilin and L-glutamin (Sigma-Aldrich, Saint Louis, MO, USA). Cells (1 × 10^6^ cells/mL) were then infected with metacyclic trypomastigotes of *T*. *cruzi* clone Dm 28c at a ratio of 5:1 parasite:cell [19]. After 1 h of incubation, macrophages and free (non-interiorized) parasites in the medium were citospun onto slides. At the end of the exposure period (24h), non-interiorized parasites were removed by repeated washings. For in vivo infection, female Holtzman rats aged 27–30 days (obtained from Universidade Federal de Minas Gerais animal facility) were inoculated intraperitoneally with of 3 X 10^5^ of *T*. *cruzi* Y strain as described elsewhere [20]. Fresh blood samples taken from the tail showed living trypomastigotes in all animals at 12 days after inoculation. At this time, animals were euthanized in a CO_2_ chamber and fragments of the heart (atria) were processed for both histopathological and ultrastructural studies as described below.

### Ethics statement

This study was carried out in full accordance with all international and Brazilian accepted guidelines and was approved by the Oswaldo Cruz Foundation Ethics Committee on Animal Use (CEUA-*Comissão de Ética no Uso de Animais*, under protocol CEUA: P-0069). CEUA follows the Brazilian national guidelines recommended by CONCEA (Conselho Nacional de Controle em Experimentação Animal). Animals were monitored daily for survival and well-being status (home cage evaluation, body condition, skin lesions, mobility and general conditions such as diarrhea). No animals died prior to the experimental endpoint (12 days of infection).

### Culture of trypomastigotes

*T*. *cruzi* Dm 28c strain was obtained and kept in the laboratory as previously described [21]. *T*. *cruzi* cultures was grown in Brain Heart Infusion medium (BHI) (BD, Franklin Lakes, NJ, USA) at 27°C in a B. O. D. (Biochemical Oxygen Demand) incubator (Thermo Scientific, Walthan, MA, USA) for 7 days, containing approximately 100% of epimastigotes. At the end of the exponential phase, parasites were centrifuged at 2200 rpm for 15 min at 10°C, resuspended in artificial triatomine urine (TAU) (190 mM NaCI, 8 mM phosphate buffer pH 6.0, 17 mM KCl, 2 mM CaCI_2_, 2 mM MgCI_2_), and incubated for 2 h at room temperature (RT). The parasites were diluted to a final concentration of 5 x 10^6^ parasites ml^-1^ in TAU supplemented with 2.5% (v/v) sodium bicarbonate 1.4%, 500 units penicillin mL^-1^, 10 mM L-proline (TAU-P medium) and incubated at 27°C in tightly closed culture flasks in a B. O. D. incubator for 5 days, obtaining approximately 100% of metacyclic trypomastigotes [22].

### Trypomastigote stimulation and viability

Trypomastigotes (1.7 x 10^6^ cells per ml) were incubated in 24 well-plates with AA (1.5–10 μM), oleic acid -OA- (1 and 5 μM), or vehicle (0.1% ethanol) for 1 or 24 h at 27°C. Subsequently, trypomastigotes (1 x 10^5^ cells per slide) were cytocentrifuged (550 rpm for 5 min) onto glass slides. Cell viability, determined by the trypan blue dye exclusion at the end of each experiment, was always greater than 90%.

### Cells and tissue preparation for TEM

Samples from cultured trypomastigotes alone were immediately fixed in a mixture of phosphate buffer 1%, pH 7.3 and freshly prepared aldehydes (1% paraformaldehyde and 1% glutaraldehyde) [23] for 1 h, at room temperature (RT), washed in the same buffer and centrifuged at 1500 g for 1 min. Samples were then re-suspended in molten 2% agar in phosphate buffer 1% and quickly re-centrifuged. To obtain optimal morphology, *T*. *cruzi*-infected macrophages directly on the slide surface were fixed as above, after 24 h of infection. Atria fragments collected from infected animals were also fixed in the same fixative for 4 h. After fixation, all samples were kept in phosphate buffer at 4°C for subsequent EM procedures.

### Histological analyses

Fragments of the heart (atria) from controls and infected animals were fixed in 4% paraformaldehyde in buffered phosphate, pH 7.3, 0.1 M for 24 h, dehydrated and embedded in plastic resin–glycol metacrilate–(Leica, Heidelberg, Germany) as previous work [24]. Semi-serial 5-μm-thick sections were cut on a microtome (RM 2155; Leica) stained by hematoxylin and eosin and examined for evaluation of the inflammatory processes and parasitism.

### TEM

Cultured trypomastigotes alone, *T*. *cruzi*-infected macrophages and heart fragments from infected animals were fixed in a mixture of freshly prepared aldehydes (1% paraformaldehyde and 1.25% glutaraldehyde) in 0.1 M sodium cacodylate buffer for 1 h (isolated cells) or 4h (tissue) at RT and processed as before [25]. Samples were post-fixed in 1% osmium tetroxide in Sym-Collidine buffer (pH 7.4) for 2 h at RT. After washing with sodium maleate buffer (pH 5.2), pellets were stained en bloc in 2% uranyl acetate in 0.05 M sodium maleate buffer (pH 6.0) for 2 h at RT and washed in the same buffer as above before dehydration in graded ethanol’s and infiltration and embedding with a propylene oxide-Epon sequence (Eponate 12 Resin; Ted Pella, Redding, CA, USA). After polymerization at 60°C for 16 h, thin sections were cut using a diamond knife on an ultra-microtome (Leica, Baden-Württemberg, Germany). Sections were mounted on uncoated 200-mesh copper grids (Ted Pella) before staining with lead citrate and viewed with a transmission electron microscope (CM 10; Philips, or Tecnai–G2-20-FEI 2006, Eindhoven, the Netherlands) at 60 kV.

### TEM quantitative analyses

In addition to qualitative observations, quantitative study was made in electron micrographs. The area and electron-density of cytoplasmic LBs within parasites were evaluated in thin sections of peritoneal and heart macrophages infected with amastigotes. LBs were classified as Strongly Electron-Dense (SED), Electron-Dense (ED) or Electron-Lucent (EL) according to a grayscale (0–85, 86–170, 171–255, respectively) where 0 is absolute black and 255 absolute white. For LB evaluation, a total of 36 electron micrographs, 50 parasites and 125 LBs were carefully analyzed. Quantitative EM analyses were also performed in electron micrographs randomly taken from metacyclic trypomastigotes to investigate the occurrence of morphological alterations. For this analysis, a total of 50 electron micrographs (25 from control and 25 from AA-stimulated) were used for evaluation of the parasite area and length. All analyses were performed using the ImageJ^®^ software (National Institutes of Health, Bethesda, MD, USA).

### LB staining

Different techniques were used for LB staining and quantification. For most studies, trypomastigotes were stained with osmium tetroxide and enumerated as before [26]. LBs within trypomastigotes were also visualized with different fluorescent probes. Cells were incubated with 1μL BODIPY493/503 dye (4,4-difluoro-1,3,5,7,8- pentamethyl-4-bora-3a,4a-diaza-s-indacene) (Molecular Probes, Eugene, OR, USA) for 1 h at 37°C or with Nile Red (9-diethylamino-5H-benzo[alpha]phenoxazine-5-one) (Sigma-Aldrich) (1/10,000 from a stock solution of 0.1 mg/mL in acetone) [26]. Alternatively, analysis of LBs were performed by incubating the cells with 0.5% Oil Red O (1-([4-(Xylylazo)xylyl]azo)-2-naphthol) (Sigma Aldrich) for 10 min at 60°C. After incubation, trypomastigotes were washed twice in Ca^2+^/Mg^2+^-free HBSS (HBSS-/-), cytospun onto slides, and fixed in 3.7% formaldehyde at RT for 10 min. Slides were mounted with VECTASHIELD^®^ mounting medium containing DAPI (4',6-Diamidino-2-Phenylindole) (Vector Laboratories, Burlingame, CA, USA) for nuclear recognition, and examined under BX-51 fluorescence microscopy and digital color camera XC-50, using a x100 objective lens (Olympus, Tokyo, Japan).

### LB purification

LB purification from control and AA-stimulated (7.5 μM trypomastigotes was performed by modifications of prior methods [27]. The trypomastigotes (1.5 x 10^9^) in 20 nM/L Tris, 1 mM/L EDTA, 1 mM/L EGTA, 100 mM/L KCl buffer (pH 7.4) containing 10 μg/mL leupeptin, 10 μg/mL benzamidin, 0.7 μg/mL pepstatin, and 0.1 mM/L phenylmethylsulfonylfluoride were disrupted by nitrogen cavitation at 700 Ψ for 5 min at 4°C. The homogenates were centrifuged at 1800 rpm for 5 min to remove the nuclei. The supernatants were overlaid sequentially with 1.5 mL each of 0.27 M/L sucrose buffer, 0.135 M/L sucrose buffer, TEE solution [25 mM/L Tris-HCl, 1 mM/L EDTA, and 1 mM/L EGTA (pH 7.4)] and centrifuged at 35,000 rpm at 4°C for 70 min. The LBs were collected from the first and second top fractions. After that, a lactate dehydrogenase activity assay was performed to demonstrate that the samples were free of cytoplasmic contaminating contents.

### Raman spectroscopy

Trypomastigotes (2 x 10^6^ cells per mL) were stimulated for 1h with 7.5 μM AA as above and centrifuged. Cells were then resuspended in 3.7% formaldehyde overnight, pelleted and analyzed by Raman spectroscopy without any labeling. Raman spectra of cells were recorded using a FT-Raman spectrometer model RFS 100S coupled to RamanScopeIII (Bruker Optik GmbH, Ettlingen, Germany), equipped with a ND:YAG laser with an excitation line at 1064 nm. For acquisition of the spectra, the laser power was adjusted to 500 mW (at source) and a good signal/noise ratio was obtained by performing 2048 scans in the region of 3500–50 cm^-1^ with a spectral resolution of 4 cm^-1^. The acquisition of Raman spectra was performed by OPUS 6.0 software (Bruker).

To investigate the presence of AA directly in LBs, purified LBs from untreated and 7.5 μM AA-treated groups were placed over 20 mm CaF_2_ windows (cat. number 63207; Edmund Optics, Barrington, NJ, USA) and the data collected with a laser power of 20 mW, 50 s integration time and 5 co-additions, without any labeling. The Raman spectra were obtained in a Senterra Raman spectrometer (Bruker) based in a 180° backscattering configuration and using a 50x objective and the 632.8 nm wavelength of He-Ne laser output as excitation. A spectral resolution of 3–5 cm^-1^ and slit width of 50x1000 μm were chosen.

### MALDI mass spectroscopy

All MALDI spectra were obtained using a time of flight mass spectrometer. Mass spectrometry matrix-assisted laser desorption/ionization-time-of-flight (MALDI-TOF) [28, 29] experiments were performed using a pulsed nitrogen laser at 337 nm of a Shimadzu Biotech Axima Performance MALDI-TOF at the Physics Department, Federal University of Juiz de Fora. The matrix was alpha-cyano-4-hydroxycinnamic acid (α-CHCA), dissolved in acetonitrile-HPLC/milli-Q water quality (50:50 v:v) at a concentration of about 5×10^−2^ mol/L. Samples of purified LBs were dissolved in phosphate buffer and 10 μL of this final sample solution were added to 10 μL of the matrix solution. This mixture (~0.5 μL) was then deposited in the stainless steel multiprobe and allowed to dry before introduction into the mass spectrometer. A typical starting laser power is 40–50. The instrument was set in the high-resolution in positive reflector ion mode and spectra were taken from 0 to 500 m/z. The experimental setup includes an automatic sample manipulator, where 200 scans were accumulated with 20 repetitions each.

### Macrophage culture and BCG *in vitro* infection

Infected macrophages were used as positive controls for Western Blotting analyses of eicosanoid-forming enzymes as below. For the in vitro infection, peritoneal macrophages from C57BL/6 mice were harvested with sterile RPMI 1640 cell culture medium. Cells (1x10^6^ cells/mL) were allowed to adhere in culture plates (6 wells) for 2 h at 37°C in a 5% CO_2_ atmosphere and were vigorously washed twice with PBS to remove nonadherent cells. Macrophages were infected with BCG (*Mycobacterium bovis* BCG (Moreau strain) vaccine from the Fundação Athaulpho de Paiva, Rio de Janeiro, Brazil), multiplicity of infection (MOI) 1:1 and incubated for 24 h at 37°C in a CO_2_ atmosphere with RPMI 1640 cell culture medium containing 2% Fetal Bovine Serum (FBS) as before [30].

### Western blotting

Macrophages (1x10^6^ cells) and trypomastigotes lysates (3x10^6^ cells) were prepared under reducing and denaturing conditions and subjected to SDS-PAGE. Samples were submitted to electrophoresis in 10% acrylamide gradient SDS-PAGE gels. After transferring onto nitrocellulose membranes, nonspecific binding sites were blocked with 5% nonfat milk in TBST (50 mM Tris-HCl (pH 7.4), 150 mM NaCl, 0.05% Tween 20). Membranes were probed with the polyclonal antibody (Ab) anti-PGE synthase (Santa Cruz Biotechnology, Dallas, TX, USA, S-16; sc-12268), anti-cyclooxygenase-2 (COX-2) (Santa Cruz Biotechnology, C-20; sc-1745) and anti-actin monoclonal Ab (BD Transduction Laboratories, 612657) in TBST with 1% nonfat dry milk. Proteins of interest were then identified by incubating the membrane with HRP-conjugated secondary Abs in TBST, followed by the detection of antigen-Ab complexes by Supersignal Chemiluminescence (GE Healthcare, Fairfield, CT, USA, ECL^™^ Prime Western Blotting System, RPN2232). A colleague blind to the identity of the sample performed the spotting and the analysis parameters.

### Immunodetection of PGE_2_ at its sites of production

Immunolocalization of PGE_2_ at its *in vivo* sites of production was performed in AA-stimulated trypomastigotes [31]. Briefly, unstimulated (kept in vehicle) and AA-stimulated (7.5 μM) cells were incubated, after 1h of stimulation, with EDAC (*N*-(3-Dimethylaminopropyl)-*N*′-ethylcarbodiimide hydrochloride) (Sigma-Aldrich), at 37°C. Besides the precise positioned coupling of an immuno-detectable eicosanoid at its sites of formation, EDAC enables: (I) the ending of cell stimulation step; (II) cell fixation; (III) cell permeabilization, allowing the penetration of both anti-eicosanoid and the detecting fluorochrome-conjugated antibodies into cells; and, importantly, (IV) the relative preservation of lipid domains, such as membranes and droplets, which dissipate with air drying or commonly used alcohol fixation [31]. Trypomastigotes were then washed with HBSS, cytospun onto glass slides, and incubated with mouse anti-PGE_2_ (1/100) Ab (Cayman Chemical—414013, Ann Arbor, MI, USA) in 0.1% normal goat serum and guinea pig polyclonal anti-mouse perilipin 2/adipose differentiation related protein (PLIN2/ADRP) Ab (Fitzgerald - 20R-AP002) (1/ 1000) in 0.1% normal donkey serum simultaneously for 1 h at RT. IgG_1_, kappa monoclonal Ab isotype control, clone MOPC 21 (Sigma Aldrich) and non immune guinea pig serum were used as controls for PGE_2_ and PLIN2/ADRP Abs, respectively. Cells were washed twice and incubated with secondary Abs, goat anti-mouse conjugated with AlexaFluor-488 (1/1000) (Molecular Probes), and CY3-conjugated donkey anti-guinea pig (1/1000) (Jackson Immuno Research Laboratories). The slides were then washed (three times, 10 min each) and mounted with Vectashield^®^ mounting medium containing DAPI (Vector Laboratories). Cells were analyzed by both phase-contrast and fluorescence microscopy. For colocalization quantitative study, fluorescence images were taken and colocalizations were quantified for 12 cells. Quantifications were determined using *ImageJ* software by calculating the Pearson’s correlation coefficient that measures the degree of linear dependence between the localization of a red signal and the localization of a green signal in the same cell [32].

### PGE_2_ evaluation

PGE_2_ levels were measured directly in the supernatant from cell-free cultures from metacyclic trypomastigotes obtained 24 h after AA (7.5 μM) stimulation. PGE_2_ was assayed in the cell-free supernatant by EIA, according to the manufacturer’s instructions (PGE_2_ enzyme- linked immunoassay (EIA) kit, Cayman Chemical).

### Statistical analysis

Data were compared using the Student-Neuman-Keuls test and expressed as mean values ± SEM (*P<0*.*05*). Analyzes were performed using the software Prism 6.01 (GraphPad software, San Diego, CA, USA).

## Results

### LBs are formed within the parasite in response to host interaction

**Fig 1:**
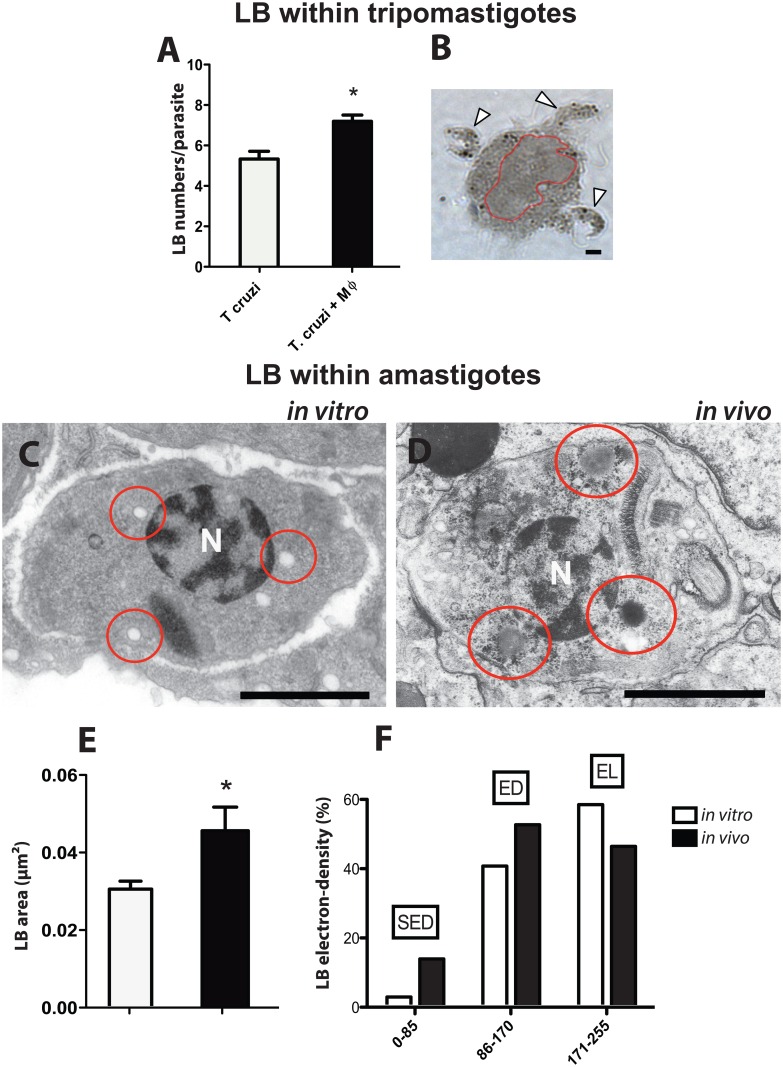
*Trypanosoma cruzi* lipid bodies (LBs) show variation in number, size and electron-density in response to the interaction with host cells. (A) Trypomastigotes cultured with peritoneal macrophages show increased numbers of cytosolic LBs compared to parasites alone, after 1h of interaction. (B) At this time, several trypomastigotes (arrows) can be seen in contact with the macrophage surface. Macrophage nucleus was outlined in red. (C, D) Transmission electron microscopy (TEM) of *T*. *cruzi* amastigotes within a peritoneal (*in vitro* infection after 24h) (C) and heart inflammatory macrophage (in vivo infection after 12 days) (D). Amastigote LBs are indicated (circles). (E) LB sizes are significantly higher in amastigotes growing in vivo (black bar) compared to amastigotes LBs living in vitro (white bar). (F) LBs from amastigote forms of the parasite within heart macrophages are more electron-dense compared to amastigote LBs from peritoneal macrophages. Cultured peritoneal macrophages and heart macrophages were fixed and processed for TEM[25]. A total of 36 electron micrographs, 50 parasites and 125 LBs were evaluated. LBs were classified as Strongly Electron-Dense (SED) Electron-Dense (ED) or Electron-Lucent (EL) by using the software ImageJ^®^ as in materials and methods. Scale bar, 10 μm (B); 5 μm (C, D). * *P<0*.*05*.

LBs are remarkably produced in host cells, mainly in macrophages, in response to *T*. *cruzi* infection [33–36]. We wondered if LBs could also be formed in the parasite cytoplasm in response to the interaction with these inflammatory cells. Metacyclic trypomastigote forms cultured in the presence of peritoneal macrophages for 1 h showed indeed a significant increase of LB numbers compared to trypomastigotes alone (Fig 1A and 1B). This indicates that the contact of *T*. *cruzi* infective forms with inflammatory host cells might modulate LB formation in the parasite.

*T*. *cruzi* grows and reproduce inside host cells, as amastigotes. Therefore, we next investigated the ultrastructure of LBs in these intracellular forms of the parasite by TEM in both peritoneal (after 24 h of the in vitro infection) and heart inflammatory macrophages (after 12 days of the acute in vivo infection). At these time points of the infection, there is consistent division of amastigotes. Moreover, the parasitism peak in the heart, a target organ of the Chagas’ disease, is observed at day 12 of the acute infection in conjunction with an elevated number of infiltrating activated macrophages [24, 37]. Histolopathological analysis of the myocardium at this time of the infection confirmed the presence of prominent mononuclear inflammatory processes and amastigote nests (S1 Fig)

LBs within amastigotes were observed as round, typical non-membrane bound organelles with varied electron-density (Fig 1C and 1D). Interestingly, LBs in amastigotes growing in vivo within macrophages infiltrated in the myocardium (Fig 1D) showed higher sizes compared to LBs formed within amastigotes living in cultured macrophages (Fig 1C) as shown by morphometric analyses (Fig 1E).

Since LBs formed within host cells such as macrophages change electron-density during inflammatory responses (reviewed in [38]), we next analyzed electron-density aspects of LBs formed within amastigotes. Based on our previous studies [34, 38, 39] and using a software for analyzing different electron-density gradations [39], LBs were classified as strongly electron-dense (SED), electron-dense (ED) or electron-lucent (EL) organelles. Quantitative TEM studies revealed that LBs from amastigotes living in heart macrophages (in vivo infection) were more electron-dense, with elevated numbers of SED and ED LBs compared to LBs from peritoneal macrophages (in vitro infection) (Fig 1F; compare Fig 1C and 1D).

Taken together, these results show that parasite LBs are not inert but dynamic organelles, able to respond to host interaction and inflammatory events.

### AA induces rapid LB formation in *T*. *cruzi* infective forms

**Fig 2:**
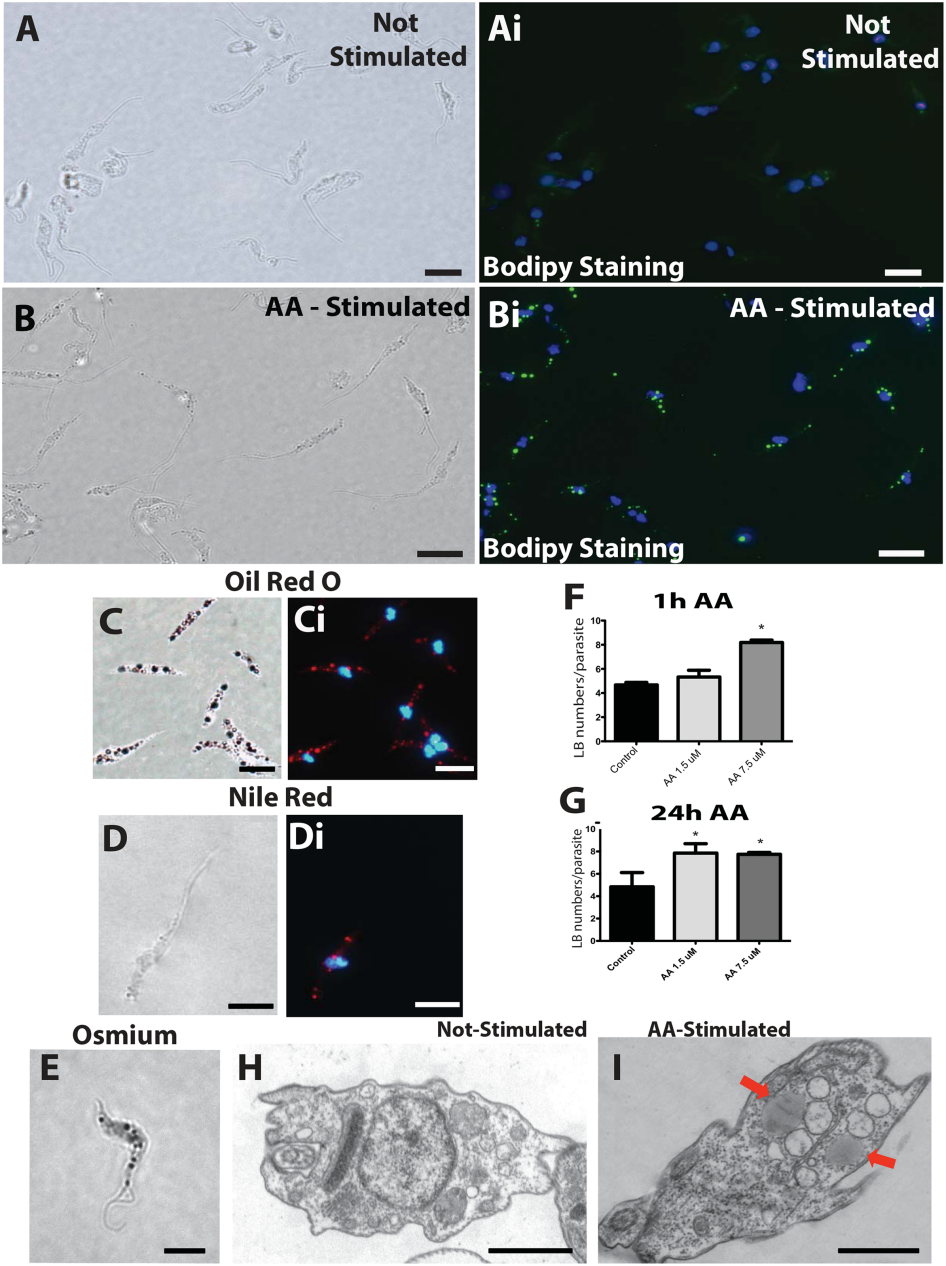
Lipid bodies (LBs) are formed within trypomastigotes in response to arachidonic acid (AA) stimulation. Metacyclic trypomastigotes were stained with BODIPY (A, Ai, B, Bi), Oil Red O (C, Ci), Nile Red (D, Di) or osmium (E) for LB detection. Panels A and Ai; B and Bi; C and Ci, and D and Di represent identical fields of trypomastigotes seen by contrast phase and fluorescence microscopy after 1 h of incubation with vehicle (A, Ai) or 7.5 μM AA (B-D). Note in (C) that Oil Red O staining enables visualization of LBs at both contrast phase and fluorescence microscopy. Parasite nuclei are visualized after DAPI staining in Ai, Bi, Ci and Di. In (E), osmium staining is observed under bright field microscopy. (F, G) Number of LBs within trypomastigotes after stimulation with exogenous AA for 1 h (F) or 24 h (G). Bars represent the mean ± SEM of LB per parasite from 50 consecutively counted parasites from at least 4 independent pools. * *P < 0*.*05* between groups. Cells were enumerated after osmium staining. In (H, I), trypomastigotes are observed by transmission electron microscopy. Typical non-membrane bound LBs (arrows) are seen in the cytoplasm after stimulation with AA (I). Scale bar, 5 μm (A-E); 1 μm (H, I).

Earlier studies demonstrated that AA is a potent stimulator of LB formation in mammalian cells [40–41] and that these organelles incorporate AA, mainly esterified in phospholipids [42–44]. The effect of AA on parasite LB formation was investigated. Four different staining techniques were used for LB visualization: osmium tetroxide, which stains phospholipids and three fluorescent probes (BODIPY, Oil Red O and Nile Red), which are more specific for neutral lipids [5, 26]. All methods enabled clear visualization of LBs within the parasite (Fig 2A–2E). Moreover, by enumerating LBs with osmium staining, it was observed a dose-dependent increase in LB numbers in AA-stimulated trypomastigotes compared to unstimulated controls (kept in vehicle) after 1 or 24 h, with a maximum LB formation at the dose of 7.5 μM (Fig 2F, 2G and S2 Fig). Trypomastigote forms of the parasite were also evaluated by TEM (Fig 2H and 2I). LBs were seen as typical, cytoplasmic non-membrane bound organelles (Fig 2I, arrows). AA had no effect on the general morphology of stimulated parasites compared to unstimulated ones as shown by qualitative and quantitative ultrastructural analyses (S3 Fig)

### AA is incorporated into LBs

**Fig 3:**
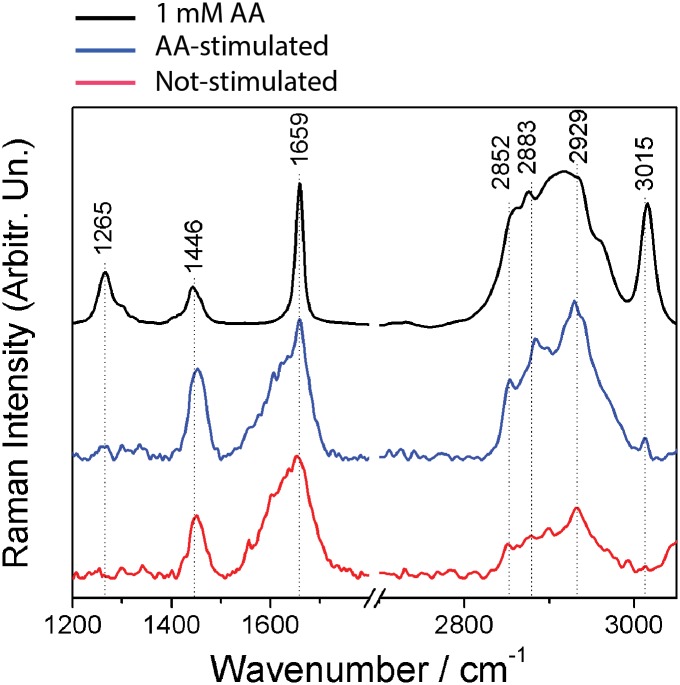
Raman spectroscopy detects higher content of unsaturated fatty acids and arachidonid acid (AA) in stimulated parasites compared to control unstimulated cells. Raman spectra of AA (black), AA-stimulated (blue) and unstimulated (red) parasites. Parasites were stimulated or not for 1h with 7.5 μM AA, fixed and analyzed by Raman spectroscopy without labeling.

To explore the molecular properties of lipids within the parasites, we next used Raman spectroscopy. This technique provides information about the structure of chemical components present in the biological sample, with the advantages of minimal sample preparation, without need of labeling and free from water interference [45]. The result is showed by the Raman spectrum, where the intensity of the signals is proportional to the relative concentration of a compound [45]. Fig 3 shows Raman spectra of AA (positive control), unstimulated and AA-stimulated parasites. The spectrum of stimulated cells differed from the spectrum of unstimulated cells by increased relative intensity of the bands at 3015 and 2929 cm^-1^, which are characteristic of AA-lipid spectra [46]. The band exhibited at 3015 cm^-1^ which corresponds to the = C-H stretching, indicates higher content of unsaturated lipids in stimulated cells (Fig 3). This band is more prominent in AA than in other fatty acids [46]. AA also has a broad and intense band at around 2920 cm^-1^ region due to C-H_2_ stretching vibrations. The observed increase in the relative intensity of the 2929 cm^-1^ in the stimulated cells may be due to the presence of the 2920 cm^-1^ broad band of AA. Therefore, the increase in the relative intensity of the bands at 3015 and 2929 cm ^-1^ are indicative of the AA incorporation. These results showed that AA is incorporated into parasites, induces LB formation and that these organelles are likely involved in the AA metabolism.

**Fig 4:**
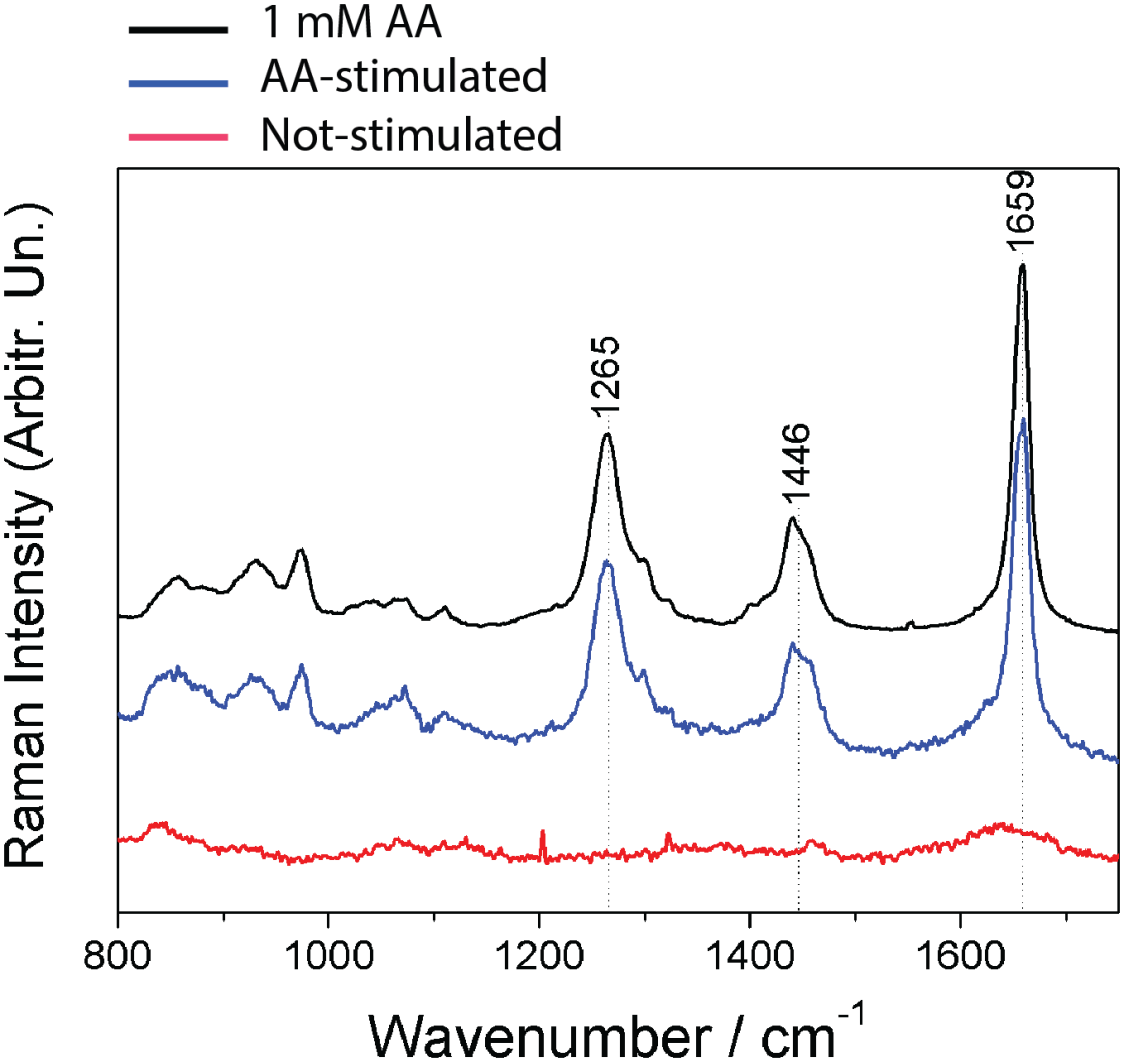
Arachidonic acid (AA) is incorporated into parasite lipid bodies (LBs). Raman spectra of 1mM AA in aqueous solution (black) and from LB fractions isolated by subcellular fractionation from unstimulated (red) and AA-stimulated (blue) parasites show that the chemical composition of LBs is due to AA and not to the presence of other fatty acids. The spectra were normalized using the band at 1659 cm^-1^. Samples were analyzed without any labeling. Data are representative of 2 independent experiments.

The Raman spectra obtained from the entire parasite have also contribution from different types of lipids and from other biomolecules, as for example proteins and DNA, which can make difficult to obtain the relative contribution of different lipids to the Raman spectra. To overcome this difficulty, we isolated LB using subcellular fractionation from unstimulated and AA-stimulated parasites and used Raman directly on LBs. This technique has been used as a valuable tool to study LBs in physiology and pathology [47]. The results are shown in Fig 4. By using this approach, it was possible to determine that the major contribution to the LB comes from the presence of AA. As shown in Fig 4, the spectrum of AA in solution is similar to the previous published in the literature [47]. It has the more intense bands at 1265 cm^-1^ (= C-H in plane bending), 1446 cm^-1^ (C-H bending) and 1659 cm^-1^ (C = C stretching) [47]. The spectrum of the non-treated LB fraction did not show any spectral characteristic of the AA (Fig 4). However, the spectra of the LB fraction obtained from parasites treated with AA showed the same spectral profile than that of the AA in solution, which demonstrate the presence of the AA in the sample (Fig 4).

**Fig 5:**
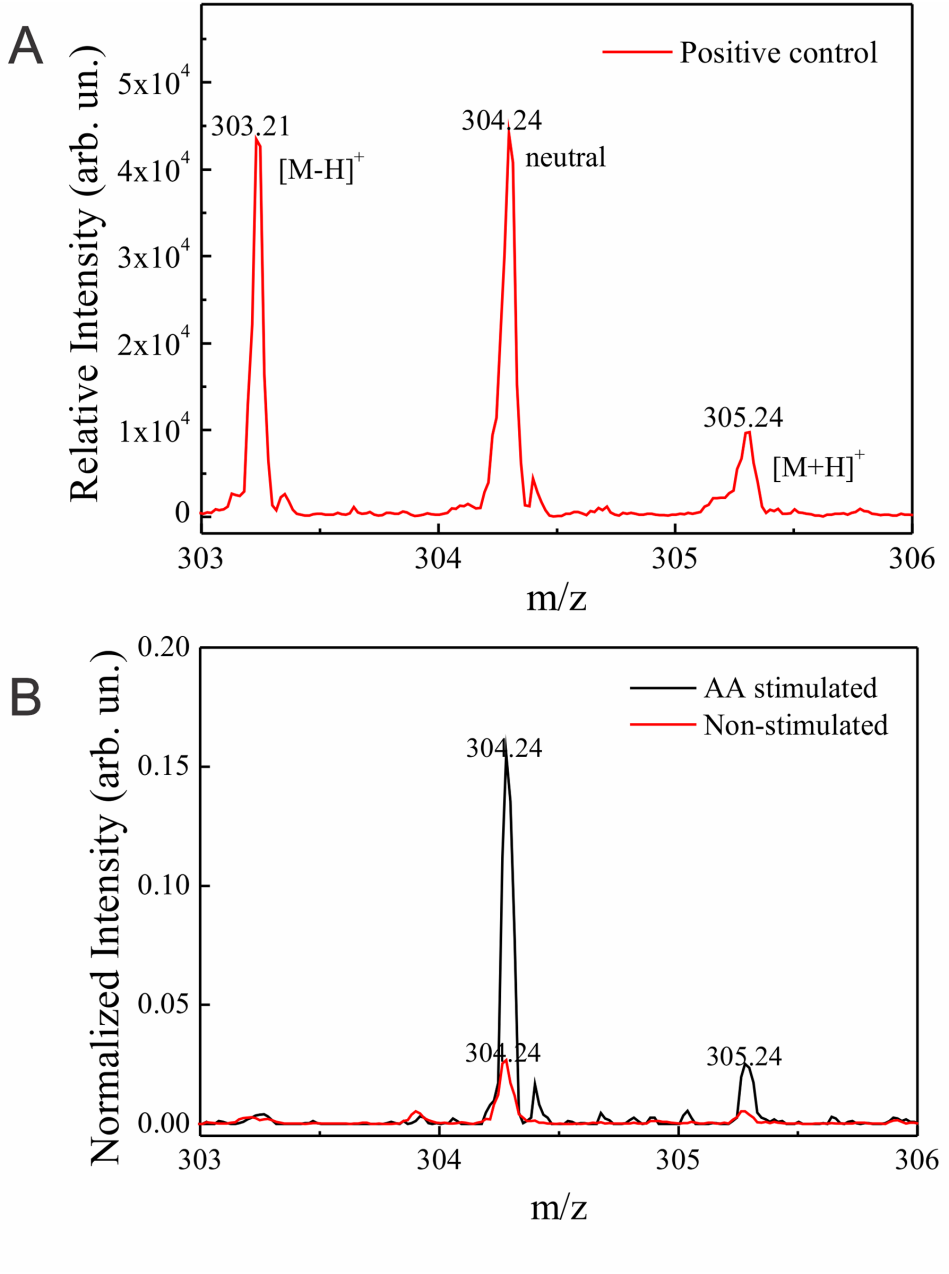
MALDI-TOF mass spectrum, from 303 to 306 m/z range, acquired from a pure arachidonic acid (AA) solution (A) and from lipid body (LB) fractions isolated from unstimulated and AA-stimulated parasites (B). In (A), the spectrum shows the molecular weight of the pure AA (m/z 304.24). In (B), a higher content of AA is observed in the LB fraction purified from stimulated parasites (black) compared to the LB fraction from unstimulated cells (red). Samples were analyzed without any labeling. Data are representative of 3 independent experiments.

The presence of increased AA in the LB fraction purified from the stimulated group was next confirmed by MALDI-TOF mass spectroscopy (Fig 5). First, a spectrum from a pure AA solution (m/z 304.24) was acquired as a positive control (Fig 5A). Second, this technique identified the m/z 304.24 peak related to the AA in both non-stimulated and AA-stimulated samples. In order to verify the existence of a fluctuation at the m/z 304.24 peak of the non-stimulated and AA-stimulated sample, the yield related to this peak was calculated by dividing the peak area by the total area of the corresponding spectrum, as before [48]. Thus, m/z 304.24 peak from the non-stimulated and AA-stimulated samples showed a related yield of 0.0035 and 0.018 respectively, that is, 5 times higher in the stimulated group (Fig 5B, black line) compared to the value obtained from the non-stimulated sample (Fig 5B, red line).

Therefore, by using different approaches, our results consistently demonstrate that LBs are cytoplasmic sites of AA accumulation in the parasite *T*. *cruzi*.

### AA-stimulated parasites generate PGE_2_

**Fig 6:**
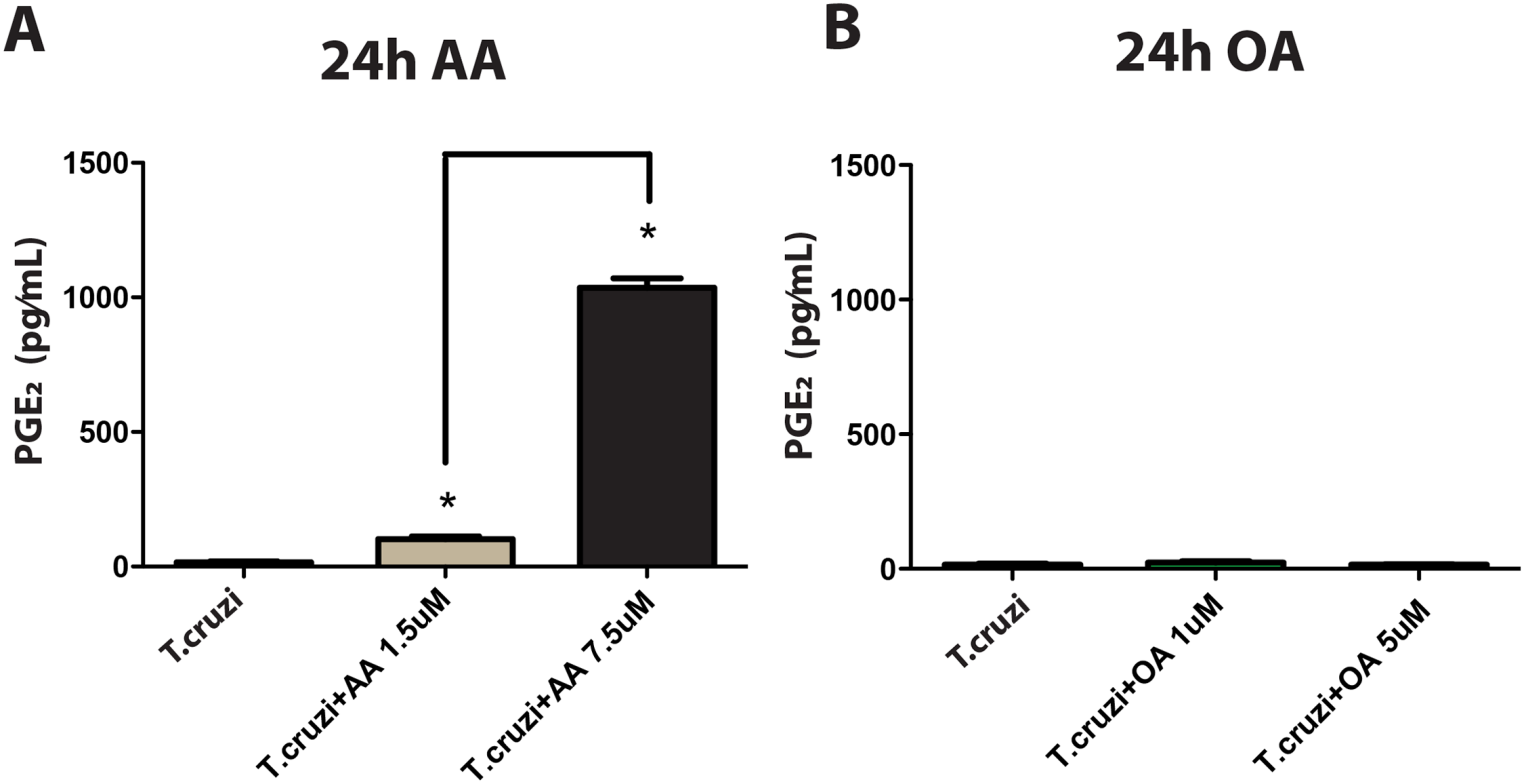
Arachidonic Acid (AA) induces prostaglandin E_2_ (PGE_2_) production by metacyclic trypomastigotes. (A) PGE_2_ levels in supernatants harvested from trypomastigotes stimulated for 24 h with 1.5 or 7.5 μM AA. (B) Oleic Acid (OA) did not induce PGE_2_ production. Bars represent the mean ± SEM, n = 3, being the graph representative of, at least, 3 independent experiments (*) *P < 0*.*05*.

Biologically active eicosanoids such as PGs are derived from AA [8, 49]. We next investigated if the parasite would produce PGs upon AA stimulation. Since PGE_2_ is consistently synthesized during *T*. *cruzi* infections [35, 50], we evaluated the production of this PG by trypomastigotes. Incubation of these parasite forms with AA produced high levels of PGE_2_ after 24h compared to controls cultured in the absence of AA (Fig 6A). Incubation of the parasites with OA, which is also able to induce LB formation in trypomastigotes (S4 Fig), did not induce PGE_2_ production (Fig 6B). Thus, we concluded that *T*. *cruzi* is capable to generate PGE_2_ through the AA metabolism.

### Parasites show PGE synthase but not COX-2 expression

**Fig 7:**
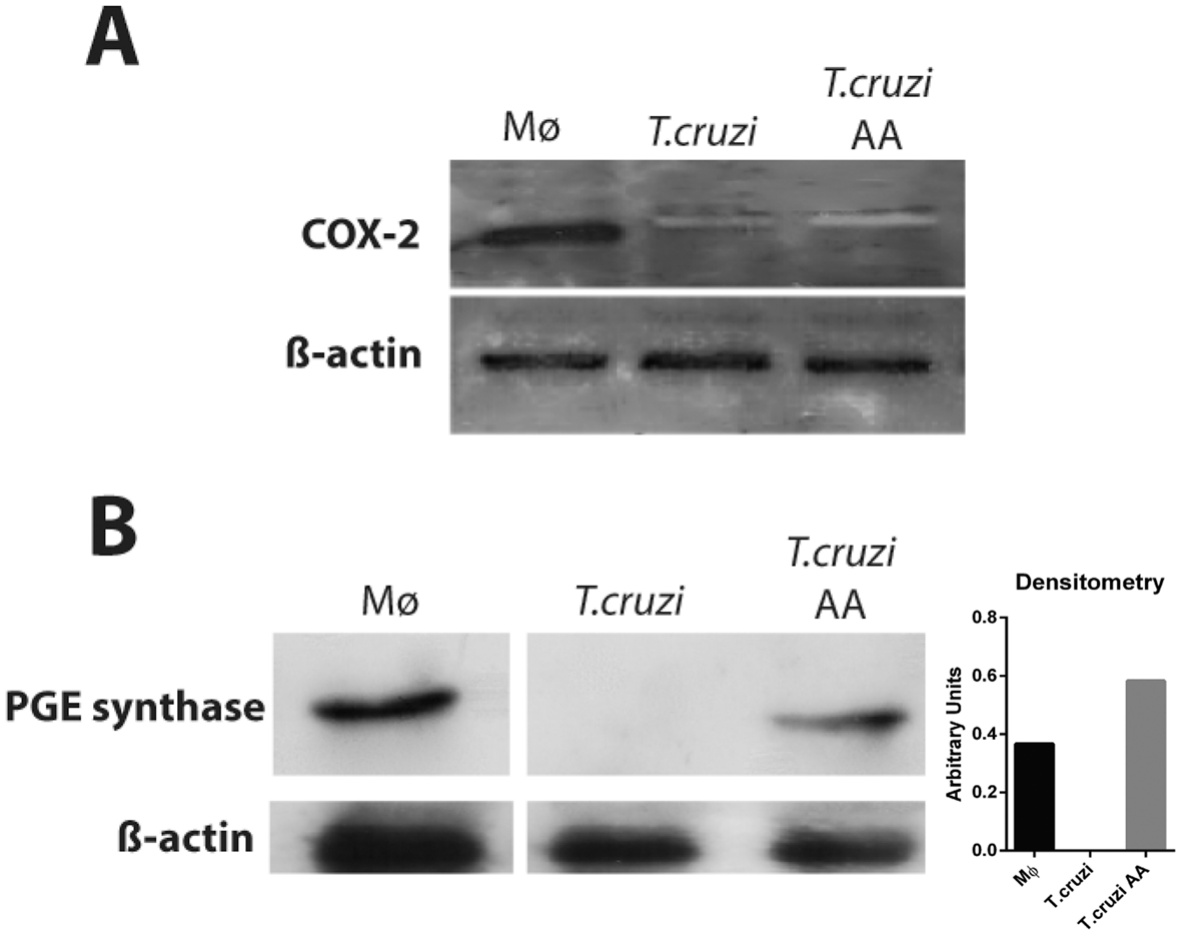
*T*. *cruzi* stimulated by AA expresses PGE synthase, but not COX-2 enzymes. Expression of COX-2 (A) and PGE synthase (B) in trypomastigote forms of *T*. *cruzi* after stimulation or not with AA during 1h. Mice peritoneal macrophages (Mϕ) stimulated by BCG during 24h were used as positive controls [30]. Total *T*. *cruzi* (2 x 10^6^ cells/lane) and macrophage cell (1 x 10^6^ cells/lane) lysates were separated by SDS-PAGE (10%) and subjected to Western blotting for COX-2, PGE synthase or β-actin. The images are representative of at least two different blots. The graph represents the densitometric analysis of PGE synthase enzyme (arbitrary units) of the Western blotting bands.

We next evaluated by Western blotting whether key eicosanoid-forming enzymes COX-2 and PGE synthase were localized within the parasites. As positive controls, we used cultures of macrophages infected with *Mycobacterium bovis* BCG, which show both expression for these enzymes and high PGE_2_ production in response to the infection [30, 51]. Trypomastigotes lysates from both unstimulated and AA-stimulated cells were negative for COX-2 (Fig 7A). On the other hand, we found a high level of PGE synthase within AA-stimulated parasites but not in unstimulated cells (Fig 7B), indicating an activation of the AA cascade and a possible pathway for PGE_2_ synthesis.

### Parasite LBs are sites for PGE_2_ synthesis

**Fig 8:**
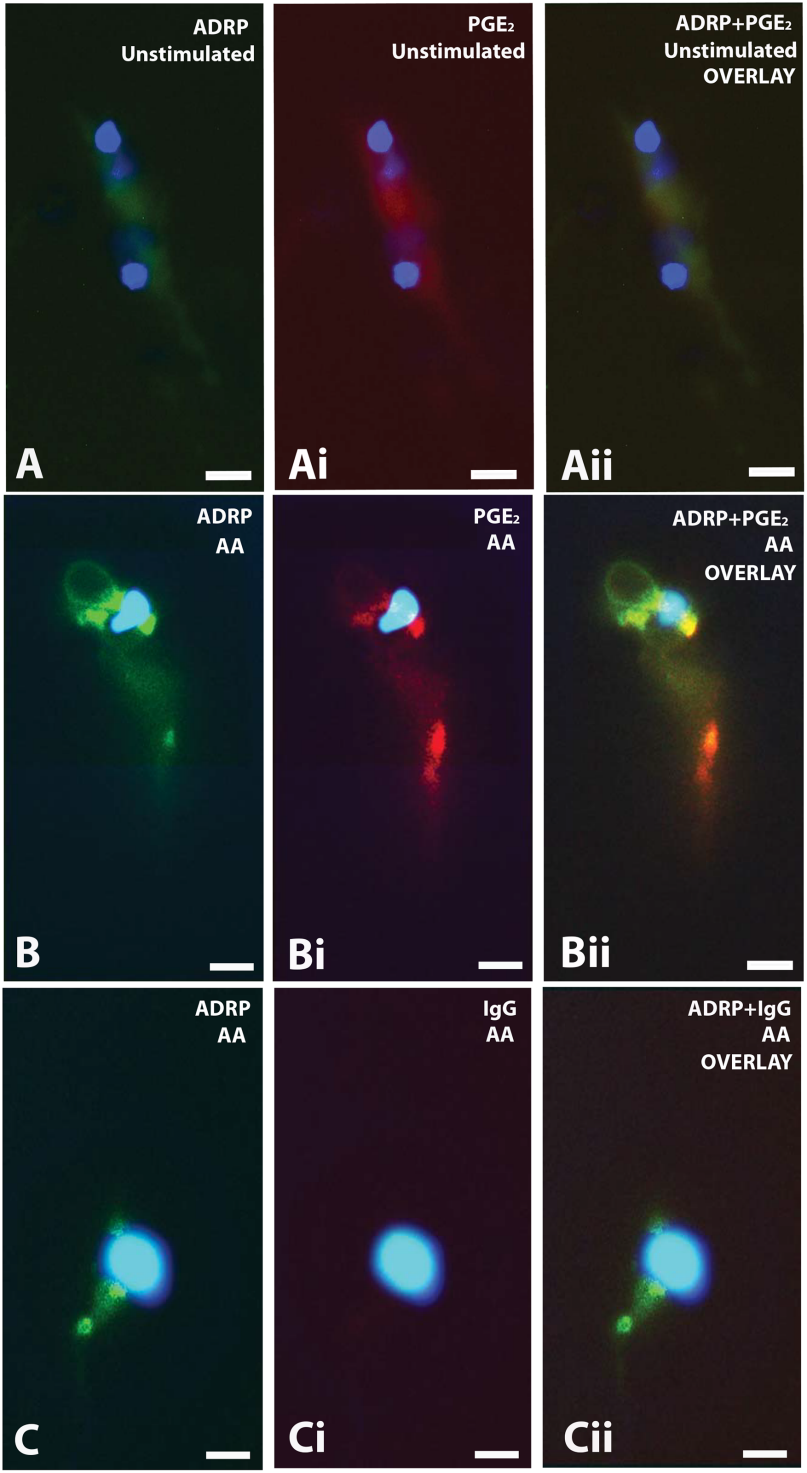
LBs are sites of PGE_2_ synthesis in AA-stimulated trypomastigotes. (A-Aii) Unstimulated trypomastigotes were labeled for PLIN2/ADRP, a marker for LBs, (A) and for PGE_2_ (Ai). Merged image (Aii) exhibited negative labeling for both ADRP and PGE_2_. (B-Bii) Arachidonic acid (AA)-stimulated trypomastigotes showed strong labeling for newly-formed PGE_2_ (Bi) in PLIN2/ADRP-associated LBs (B). The merged image is observed in (Bii). (C-Cii) An IgG1 irrelevant isotype (clone MOPC 21) was used as control for PGE_2_ labeling in combination with labeling for PLIN2/ADRP. (Cii) is the merged image of (C) and (Ci). The nuclei of parasites are observed after DAPI staining (blue). Cells were immunolabeled by using the EicosaCell technique [31]. Colocalization quantitative analyses showed that PGE_2_ and PLIN2/ADRP significantly colocalized in cells [Pearson’s correlation coefficient of 0.84 ± 0.02 (mean ± SEM)]. Scale bar, 2 μm (all images). Images are representative of 3 independent experiments.

Because parasite stimulation with AA led to quantitative increases in LB numbers, PGE_2_ release and PGE synthase expression, we hypothesized that parasite LBs might also serve as domains for compartmentalized PGE_2_ synthesis. With the adaptation to a method using carbodiimide to immobilize eicosanoid carboxyl groups to proximate proteins [52], formation of eicosanoids was investigated at its sites of production. PLIN 2/ADRP was used as a marker of LBs for co-localization purpose [31]. With the use of this technique (EicosaCell) [31], PGE_2_ formation was demonstrated specifically at LBs from trypomastigotes of *T*. *cruzi* stimulated with AA (Fig 8). AA-stimulated trypomastigotes, exhibited a strong localized punctuated or ring shape staining for PLIN2/ADRP-labeled LBs (compare Fig 8A–8C), indicating formation of LBs in response to the stimulation of AA. AA-stimulated trypomastigotes, but not vehicle-stimulated parasites, showed intense and punctuated immunofluorescent staining for PGE_2_ (compare Fig 8Ai and 8Bi). As shown in Fig 8Bii, the PGE_2_ intracellular site of production (8Bi) matched PLIN2/ADRP-stained LBs (Fig 8B). Our colocalization quantitative analyses using *ImageJ* showed that PGE_2_ and PLIN2/ADRP significantly colocalized in cells [Pearson’s coefficient of 0.84 ± 0.02 (mean ± SEM)]. The specificity of the immunofluorescence for PGE_2_ was supported by the absence of immunostaining when an isotype control antibody replaced the anti-PGE_2_ monoclonal antibody (Fig 8C–8Cii). Controls in which the anti-PLIN2/ADRP Ab was replaced by guinea pig serum were negative (data not shown). These findings validated the specificity for detecting PGE_2_ formed at its formation sites within stimulated trypomastigotes, and place LBs as candidate sites for newly formed PGE_2_ during the *T*. *cruzi* infection.

## Discussion and Conclusions

Here we demonstrate for the first time that the parasite *T*. *cruzi* itself produces LBs in response to the host-parasite interaction and that these organelles may be sources of inflammation mediators. Analogous to LB formed in mammalian leukocytes and macrophages during infectious diseases [30, 33–35, 53], increases in parasite LB numbers and sizes, in conjunction with changes in LB electron-density highlight the fact that parasite LBs are also dynamic and active organelles, able to modify their structure in concert with cell activation.

Evidence that LBs participate in the regulation of the *T*. *cruzi* response during host-parasite interaction was provided here during both in vitro and in vivo infection. Interestingly, LBs in amastigote forms growing in macrophages elicited from the in vivo infection during the acute phase showed higher size and electron-density compared to amastigotes LBs living in cultured macrophages after 1h. This observation is likely consequence of the amplified exposure of proliferating *T*. *cruzi* forms to the host inflammatory milieu. In mammalian cells, the size and electron-density of newly formed LBs represent an important structural indicative of the participation of these organelles in innate immune responses [34].

LB morphological changes may reflect differences in lipid composition, stages of formation of new LBs, and/or neutral lipids/phospholipids ratio within LBs [34]. In fact, lipid composition affects LB electron-density [54] based on the fact that osmium tetroxide binds preferentially to the unsaturated bonds of fatty acids [55, 56]. Here, we demonstrated by Raman spectroscopy that both AA-stimulated parasites and LBs isolated from AA-stimulated-parasites have a higher content of unsaturated fatty acids, as a result of AA incorporation, compared to unstimulated cells (Figs 3 and 4). The presence of increased AA in the LB fraction was also confirmed by MALDI-TOF mass spectroscopy in LBs purified from the stimulated group (Fig 5). This can explain the changes of LB electron-density within the parasites during the in vivo infection. These LBs, formed as a result of host interaction, are more electron-dense which likely reflects the high content of AA, captured by the reaction with osmium during sample preparation for TEM. Accordingly, in macrophages, increase in LB electron-density was also associated with the cascade of events involved in the synthesis of inflammatory mediators within LBs formed in response to cell activation (reviewed in [38]). Under stimulation, AA is released from its sterified pool and acts as a substrate for enzymatic conversion into lipid mediators [49].

Based on our previous findings of production of AA-derived PGE_2_ within LBs from macrophages taking part in immune responses during *T*. *cruzi* infection (reviewed in [36]), we considered whether parasite LBs might also play a role in inflammation. Evidence for prostaglandin production in parasites including *T*. *cruzi*, *T*. *brucei* and Leishmania species was provided by studies from Kubata and other groups (reviewed in [57]). Parasite enzymes involved in the AA cascade and PG synthesis were also identified in protozoans [57]. However, these studies did not associate eicosanoid synthesis with LB formation within the parasite.

Both mammalian [42, 43] and parasite [58, 59] LBs incorporate AA, the common precursor of eicosanoids. As noted, by using Raman spectroscopy, we detected AA incorporation into stimulated parasites (Fig 3). This technique also detected increased AA content directly in LBs isolated from AA-stimulated parasites (Fig 4). In fact, Raman has gained recognition for biomedical applications [60] and has been recently used to characterize molecular details associated with lipid metabolism and LB formation in macrophages [61] and host-parasite interaction [62]. Our results also demonstrated the existence of a pathway associated with the AA cascade. PGE_2_ synthase, but not COX-2, was consistently identified, for the first time, within the parasite (Fig 4). The lack of COX expression might be explained by the absence of homologs of mammalian COX in parasitic microbes although a COX-like enzyme has been reported (reviewed in [57]). On the other hand, several PG synthases with different degrees of homology with human have been identified in parasites [57].

In a recent work it was demonstrated that AA stimulation increases both the number of LBs and the release of PGF2α by metacyclic forms of the parasite *Leishmania infantum chagasi* [14]. Moreover, PGF synthase was localized in LBs formed within this parasite, thus suggesting that these organelles are sites involved in the synthesis of PGF_2_α [14].

Here, we provide direct evidence that parasite LBs are indeed intracellular sites of eicosanoid production. AA-, but not OA-stimulated trypomastigotes released PGE_2_ in parallel to a consistent formation of cytoplasmic LBs (Fig 2) and this PG was fully immunolocalized within parasite LBs (Fig 8). Our data are in agreement with a previous work demonstrating high levels of PGE_2_ in lysates of *T*. *cruzi* when incubated with AA [63]. However, the results presented here are the first association of the PG production with specific intracellular sites, i.e., LBs.

Therefore, from our present results in *T*. *cruzi* and evidences provided by studies in other protozoan parasites, as noted, it seems evident that these organisms are able to generate PGs, but what is the role of parasite-derived PGs in the pathogenesis of parasitic diseases? Because PGE_2_ is a potent immunomodulator, it could contribute to the immunosuppression observed during *T*. *cruzi* infections with implications to the survival of the parasite in its host. Investigations need to be done to explore this possibility and to uncover the PG metabolic pathways in this parasite.

In conclusion, this work demonstrates that LBs are formed in the parasite *T*. *cruzi* in response to the host-parasite interaction and exogenous AA stimulation, that AA is incorporated into parasite LBs and that these organelles serve as sites for PGE_2_ synthesis, implying a role for *T*. *cruzi*-derived PGs in Chagas’ disease pathogenesis.

## Supporting Information

S1 Fig Myocarditis elicited by the acute phase of experimental Chagas' disease. (A, B) Histopathological analyses from control (A) and infected animals show myocarditis predominantly mononuclear and diffuse, dissociated myocardial fibers and edematous intersticial tissue. Parasite nests are encircled. Holtzman rats were infected with *Trypanosoma cruzi* (Y strain) and fragments of the heart processed for histological analyses at day 12 of infection. Data are representative of, at least, 3 independent experiments. Slides were stained with hematoxylin and eosin.
(EPS)

S2 Fig Arachidonic-acid (AA)-induced lipid body (LB) formation is a rapid and dose-dependent phenomenon within the parasite *Trypanosoma cruzi*. The dose-response curve of LB genesis was analyzed 1 h after stimulation with AA (1–10 μm). LBs were visualized and enumerated using osmium staining. Results were expressed as mean ± SEM, from at least 3 experiments.
(TIF)

S3 Fig Quantitative ultrastructural analyses of the parasite *Trypanosoma cruzi* stimulated or not with 7.5 μM arachidonic acid (AA). Samples from unstimulated AA-stimulated parasites were fixed and processed for transmission electron microscopy. A total of 50 electron micrographs (25 from unstimulated and 25 from AA-stimulated groups) were analyzed and the parasite area and length were quantitated using the *ImageJ*^®^ software.
(EPS)

S4 Fig Number of lipid bodies (LBs) within trypomastigotes after stimulation with exogenous oleic acid (OA) for 1 h (A) or 24 h (B). Bars represent the mean ± SEM of LBs per parasite from 50 consecutevely counted parasites from at least 4 independent experiments. * *P* < 0.05 between groups. Cells were enumerated using osmium staining.
(TIF)

## Abbreviations

LB: lipid body
AA: arachidonic acid
OA: oleic acid
PGE_2_: prostaglandin E_2_
COX-2: cyclooxygenase 2
PLIN2/ADRP: perilipin 2/adipose differentiation-related protein
TEM: transmission electron microscopy

## References

[1] Murphy DJ. The dynamic roles of intracellular lipid droplets: from archaea to mammals. Protoplasma. 2012;249(3):541–85. Epub 2011/10/18. 10.1007/s00709-011-0329-7 . 22002710

[2] Beller M, Thiel K, Thul PJ, Jackle H. Lipid droplets: a dynamic organelle moves into focus. FEBS Lett. 2010;584(11):2176–82. Epub 2010/03/23. 10.1016/j.febslet.2010.03.022 . 20303960

[3] Fujimoto T, Ohsaki Y, Cheng J, Suzuki M, Shinohara Y. Lipid droplets: a classic organelle with new outfits. Histochem Cell Biol. 2008;130(2):263–79. Epub 2008/06/12. 10.1007/s00418-008-0449-0 18546013 PMC2491702

[4] Melo RCN, Weller PF. Unraveling the complexity of lipid body organelles in human eosinophils. J Leukoc Biol. 2014;96(5):703–12. Epub 2014/09/12. 10.1189/jlb.3RU0214-110R 25210147 PMC4197557

[5] Melo RCN, D'Avila H, Wan HC, Bozza PT, Dvorak AM, Weller PF. Lipid bodies in inflammatory cells: structure, function, and current imaging techniques. J Histochem Cytochem. 2011;59(5):540–56. Epub 2011/03/25. 10.1369/0022155411404073 21430261 PMC3201176

[6] Saka HA, Valdivia R. Emerging roles for lipid droplets in immunity and host-pathogen interactions. Annu Rev Cell Dev Biol. 2012;28:411–37. Epub 2012/05/15. 10.1146/annurev-cellbio-092910-153958 . 22578141

[7] Bozza PT, Bakker-Abreu I, Navarro-Xavier RA, Bandeira-Melo C. Lipid body function in eicosanoid synthesis: an update. Prostaglandins Leukot Essent Fatty Acids. 2011;85(5):205–13. Epub 2011/05/14. 10.1016/j.plefa.2011.04.020 . 21565480

[8] Melo RCN, Weller PF. Lipid droplets in leukocytes: Organelles linked to inflammatory responses. Exp Cell Res. 2016;340(2):193–7. Epub 2015/10/31. 10.1016/j.yexcr.2015.10.028 26515551 PMC4744558

[9] Melo RCN, Dvorak AM. Lipid Body-Phagosome Interaction in Macrophages during Infectious Diseases: Host Defense or Pathogen Survival Strategy? PLoS Pathog. 2012;8(7):e1002729 Epub 2012/07/14. 10.1371/journal.ppat.1002729 22792061 PMC3390411

[10] Herker E, Ott M. Emerging role of lipid droplets in host/pathogen interactions. J Biol Chem. 2012;287(4):2280–7. Epub 2011/11/18. 10.1074/jbc.R111.300202 22090026 PMC3268388

[11] Jackson KE, Klonis N, Ferguson DJ, Adisa A, Dogovski C, Tilley L. Food vacuole-associated lipid bodies and heterogeneous lipid environments in the malaria parasite, Plasmodium falciparum. Mol Microbiol. 2004;54(1):109–22. Epub 2004/10/02. . 15458409 10.1111/j.1365-2958.2004.04284.x

[12] Nishikawa Y, Quittnat F, Stedman TT, Voelker DR, Choi JY, Zahn M, et al Host cell lipids control cholesteryl ester synthesis and storage in intracellular Toxoplasma. Cell Microbiol. 2005;7(6):849–67. Epub 2005/05/13. 10.1111/j.1462-5822.2005.00518.x . 15888087

[13] Daniel J, Maamar H, Deb C, Sirakova TD, Kolattukudy PE. Mycobacterium tuberculosis uses host triacylglycerol to accumulate lipid droplets and acquires a dormancy-like phenotype in lipid-loaded macrophages. PLoS Pathog. 2011;7(6):e1002093 Epub 2011/07/07. 10.1371/journal.ppat.1002093 21731490 PMC3121879

[14] Araujo-Santos T, Rodriguez NE, Moura-Pontes S, Dixt UG, Abanades DR, Bozza PT, et al Role of prostaglandin F2alpha production in lipid bodies from *Leishmania infantum chagasi*: insights on virulence. J Infect Dis. 2014;210(12):1951–61. Epub 2014/05/23. 10.1093/infdis/jiu299 . 24850789 PMC6281352

[15] Mackey TK, Liang BA, Cuomo R, Hafen R, Brouwer KC, Lee DE. Emerging and reemerging neglected tropical diseases: a review of key characteristics, risk factors, and the policy and innovation environment. Clin Microbiol Rev. 2014;27(4):949–79. Epub 2014/10/04. 10.1128/CMR.00045-14 25278579 PMC4187634

[16] Tilley SL, Coffman TM, Koller BH. Mixed messages: modulation of inflammation and immune responses by prostaglandins and thromboxanes. J Clin Invest. 2001;108(1):15–23. Epub 2001/07/04. 10.1172/JCI13416 11435451 PMC209346

[17] Michelin MA, Silva JS, Cunha FQ. Inducible cyclooxygenase released prostaglandin mediates immunosuppression in acute phase of experimental *Trypanosoma cruzi* infection. Exp Parasitol. 2005;111(2):71–9. Epub 2005/07/13. 10.1016/j.exppara.2005.05.001 . 16009364

[18] Walker C, Kristensen F, Bettens F, deWeck AL. Lymphokine regulation of activated (G1) lymphocytes. I. Prostaglandin E2-induced inhibition of interleukin 2 production. J Immunol. 1983;130(4):1770–3. Epub 1983/04/01. . 6601136

[19] Luna-Gomes T, Filardy AA, Rocha JD, Decote-Ricardo D, LaRocque-de-Freitas IF, Morrot A, et al Neutrophils increase or reduce parasite burden in *Trypanosoma cruzi*-infected macrophages, depending on host strain: role of neutrophil elastase. PLoS One. 2014;9(3):e90582 Epub 2014/03/07. 10.1371/journal.pone.0090582 24599360 PMC3944110

[20] Melo RCN, Machado CRS. *Trypanosoma cruzi*: peripheral blood monocytes and heart macrophages in the resistance to acute experimental infection in rats. Exp Parasitol. 2001;97(1):15–23. Epub 2001/02/24. 10.1006/expr.2000.4576 . 11207110

[21] Contreras VT, Morel CM, Goldenberg S. Stage specific gene expression precedes morphological changes during *Trypanosoma cruzi* metacyclogenesis. Mol Biochem Parasitol. 1985;14(1):83–96. Epub 1985/01/01. . 3885031 10.1016/0166-6851(85)90108-2

[22] Contreras VT, Salles JM, Thomas N, Morel CM, Goldenberg S. In vitro differentiation of T*rypanosoma cruzi* under chemically defined conditions. Mol Biochem Parasitol. 1985;16(3):315–27. Epub 1985/09/01. . 3903496 10.1016/0166-6851(85)90073-8

[23] Melo RCN. Depletion of immune effector cells induces myocardial damage in the acute experimental *Trypanosoma cruz*i infection: ultrastructural study in rats. Tissue Cell. 1999;31(3):281–90. Epub 1999/09/11. 10.1054/tice.1999.0040 . 10481300

[24] Fabrino DL, Leon LL, Genestra M, Parreira GG, Melo RCN. Rat models to investigate host macrophage defense against *Trypanosoma cruzi*. J Innate Immun. 2011;3(1):71–82. Epub 2010/11/06. 10.1159/000320641 . 21051863

[25] Melo RCN, Spencer LA, Perez SA, Neves JS, Bafford SP, Morgan ES, et al Vesicle-mediated secretion of human eosinophil granule-derived major basic protein. Lab Invest. 2009;89(7):769–81. Epub 2009/04/29. 10.1038/labinvest.2009.40 19398958 PMC2702460

[26] Melo RCN, D'Avila H, Bozza PT, Weller PF. Imaging lipid bodies within leukocytes with different light microscopy techniques. Methods Mol Biol. 2011;689:149–61. Epub 2010/12/15. 10.1007/978-1-60761-950-5_9 . 21153791 PMC3659330

[27] Brasaemle DL, Wolins NE. Isolation of lipid droplets from cells by density gradient centrifugation. Curr Protoc Cell Biol. 2006;Chapter 3:Unit 3 15 10.1002/0471143030.cb0315s29 . 18228483

[28] Karas M, Bachmann D, Hillenkamp F. Influence of the wavelength in high-irradiance ultraviolet-laser desorption mass-spectrometry of organic-molecules. Anal Chemistry. 1985;57(14):2935–9. 10.1021/ac00291a042

[29] Karas M, Bachman K, Bahr U, Hillenkamp F. Matrix-assisted ultraviolet-laser desorption of nonvolatile compounds. Int J Mass Spectrom Ion Processes.1987;78:53–68. 10.1016/0168-1176(87)87041-6

[30] D'Avila H, Melo RCN, Parreira GG, Werneck-Barroso E, Castro-Faria-Neto HC, Bozza PT. *Mycobacterium bovis* bacillus Calmette-Guerin induces TLR2-mediated formation of lipid bodies: intracellular domains for eicosanoid synthesis in vivo. J Immunol. 2006;176(5):3087–97. Epub 2006/02/24. 176/5/3087. . 16493068 10.4049/jimmunol.176.5.3087

[31] Bandeira-Melo C, Weller PF, Bozza PT. EicosaCell—an immunofluorescent-based assay to localize newly synthesized eicosanoid lipid mediators at intracellular sites. Methods Mol Biol. 2011;689:163–81. Epub 2010/12/15. 10.1007/978-1-60761-950-5_10 . 21153792 PMC3679533

[32] Bolte S, Cordelieres FP. A guided tour into subcellular colocalization analysis in light microscopy. J Microsc. 2006;224(Pt 3):213–32. Epub 2007/01/11. 10.1111/j.1365-2818.2006.01706.x . 17210054

[33] Melo RCN, D'Avila H, Fabrino DL, Almeida PE, Bozza PT. Macrophage lipid body induction by Chagas disease in vivo: putative intracellular domains for eicosanoid formation during infection. Tissue Cell. 2003;35(1):59–67. Epub 2003/02/19. 10.1016/S0040-8166(02)00105-2 . 12589730

[34] Melo RCN, Fabrino DL, Dias FF, Parreira GG. Lipid bodies: Structural markers of inflammatory macrophages in innate immunity. Inflamm Res. 2006;55(8):342–8. Epub 2006/09/16. 10.1007/s00011-006-5205-0 . 16977381

[35] D'Avila H, Freire-de-Lima CG, Roque NR, Teixeira L, Barja-Fidalgo C, Silva AR, et al Host cell lipid bodies triggered by *Trypanosoma cruzi* infection and enhanced by the uptake of apoptotic cells are associated with prostaglandin E generation and increased parasite growth. J Infect Dis. 2011;204(6):951–61. Epub 2011/08/19. 10.1093/infdis/jir432 . 21849292

[36] D'Avila H, Toledo DA, Melo RCN. Lipid bodies: inflammatory organelles implicated in host-*Trypanosoma cruzi* interplay during innate immune responses. Mediators Inflamm. 2012;2012:478601 Epub 2012/05/24. 10.1155/2012/478601 22619483 PMC3350868

[37] Melo RCN. Acute heart inflammation: ultrastructural and functional aspects of macrophages elicited by *Trypanosoma cruzi* infection. J Cell Mol Med. 2009;13(2):279–94. Epub 2008/07/16. 10.1111/j.1582-4934.2008.00388.x . 18624767 PMC3823355

[38] Dias FF, Zarantonello VC, Parreira GG, Chiarini-Garcia H, Melo RCN. The Intriguing ultrastructure of lipid body organelles within activated macrophages. Microsc Microanal. 2014:1–10. Epub 2014/05/03. 10.1017/S143192761400066X . 24786359

[39] Dias FF, Chiarini-Garcia H, Parreira GG, Melo RCN. Mice spermatogonial stem cells transplantation induces macrophage migration into the seminiferous epithelium and lipid body formation: high-resolution light microscopy and ultrastructural studies. Microsc Microanal. 2011:1–13. Epub 2011/11/04. 10.1017/S1431927611012098 . 22047748

[40] Weller PF, Ryeom SW, Picard ST, Ackerman SJ, Dvorak AM. Cytoplasmic lipid bodies of neutrophils: formation induced by cis-unsaturated fatty acids and mediated by protein kinase C. J Cell Biol. 1991;113(1):137–46. Epub 1991/04/01. 1901065 10.1083/jcb.113.1.137 PMC2288908

[41] Bozza PT, Payne JL, Morham SG, Langenbach R, Smithies O, Weller PF. Leukocyte lipid body formation and eicosanoid generation: cyclooxygenase-independent inhibition by aspirin. Proc Natl Acad Sci U S A. 1996;93(20):11091–6. Epub 1996/10/01. 8855314 10.1073/pnas.93.20.11091 PMC38289

[42] Weller PF, Dvorak AM. Arachidonic acid incorporation by cytoplasmic lipid bodies of human eosinophils. Blood. 1985;65(5):1269–74. Epub 1985/05/01. . 3922452

[43] Weller PF, Monahan-Earley RA, Dvorak HF, Dvorak AM. Cytoplasmic lipid bodies of human eosinophils. Subcellular isolation and analysis of arachidonate incorporation. Am J Pathol. 1991;138(1):141–8. Epub 1991/01/01. 1846262 PMC1886053

[44] Yu W, Bozza PT, Tzizik DM, Gray JP, Cassara J, Dvorak AM, et al Co-compartmentalization of MAP kinases and cytosolic phospholipase A2 at cytoplasmic arachidonate-rich lipid bodies. Am J Pathol. 1998;152(3):759–69. Epub 1998/03/21. 9502418 PMC1858398

[45] Manoharan R, Wang Y, Feld MS. Histochemical analysis of biological tissues using Raman spectroscopy. Spectrochimica Acta Part A. 1996:215–49. 10.1016/0584-8539(95)01573-6

[46] Czamara K, Majzner K, Pacia MZ, Kochan K, Kaczor A, Baranska M. Raman spectroscopy of lipids: a review. J Raman Spect. 2015;46:4–20. 10.1002/jrs.4607

[47] Majzner K, Kochan K, Kachamakova-Trojanowska N, Maslak E, Chlopicki S, Baranska M. Raman imaging providing insights into chemical composition of lipid droplets of different size and origin: in hepatocytes and endothelium. Anal Chem. 2014;86(13):6666–74. 10.1021/ac501395g . 24936891

[48] Martins JS, Borges BGAL, Machado RC, Carpanez AG, Grazul RM, Zappa F, et al Evaluation of chemical kinetics in positive photoresists using laser desorption ionization. Eur Polymer J,. 2014;59:1–17. 10.1016/j.eurpolymj.2014.07.005

[49] Yaqoob P. Fatty acids as gatekeepers of immune cell regulation. Trends Immunol. 2003;24(12):639–45. Epub 2003/12/03. S1471490603003077. . 14644137 10.1016/j.it.2003.10.002

[50] Abdalla GK, Faria GE, Silva KT, Castro EC, Reis MA, Michelin MA. *Trypanosoma cruzi*: the role of PGE2 in immune response during the acute phase of experimental infection. Exp Parasitol. 2008;118(4):514–21. Epub 2008/01/01. 10.1016/j.exppara.2007.11.003 . 18163990

[51] Shibata Y, Henriksen RA, Honda I, Nakamura RM, Myrvik QN. Splenic PGE2-releasing macrophages regulate Th1 and Th2 immune responses in mice treated with heat-killed BCG. J Leukoc Biol. 2005;78(6):1281–90. Epub 2005/10/06. 10.1189/jlb.0605321 . 16204627

[52] Liu LX, Buhlmann JE, Weller PF. Release of prostaglandin E2 by microfilariae of *Wuchereria bancrofti* and *Brugia malayi*. Am J Trop Med Hyg. 1992;46(5):520–3. Epub 1992/05/01. . 1599045 10.4269/ajtmh.1992.46.520

[53] Toledo DAM, D'Avila H, Melo RCN. Host Lipid Bodies as Platforms for Intracellular Survival of Protozoan Parasites. Front Immunol. 2016 10.3389/fimmu.2016.00174 . 27199996 PMC4853369

[54] Cheng J, Fujita A, Ohsaki Y, Suzuki M, Shinohara Y, Fujimoto T. Quantitative electron microscopy shows uniform incorporation of triglycerides into existing lipid droplets. Histochem Cell Biol. 2009;132(3):281–91. Epub 2009/06/27. 10.1007/s00418-009-0615-z . 19557427

[55] Hayes TL, Lindgren FT, Gofman JW. A Quantitative Determination of the Osmium Tetroxide-Lipoprotein Interaction. J Cell Biol. 1963;19:251–5. Epub 1963/10/01. 14069797 10.1083/jcb.19.1.251 PMC2106851

[56] Adams CW, Abdulla YH, Bayliss OB. Osmium tetroxide as a histochemical and histological reagent. Histochemie Histochemistry Histochimie. 1967;9(1):68–77. Epub 1967/01/01. . 4869962 10.1007/BF00281808

[57] Kubata BK, Duszenko M, Martin KS, Urade Y. Molecular basis for prostaglandin production in hosts and parasites. Trends Parasitol. 2007;23(7):325–31. Epub 2007/05/29. 10.1016/j.pt.2007.05.005 . 17531535

[58] Longworth DL, Monahan-Earley RA, Dvorak AM, Weller PF. *Brugia malayi*: arachidonic acid uptake into lipid bodies of adult parasites. Exp Parasitol. 1988;65(2):251–7. Epub 1988/04/01. . 3350105 10.1016/0014-4894(88)90129-4

[59] Longworth DL, King DC, Weller PF. Rapid uptake and esterification of arachidonic acid and other fatty acids by microfilariae of *Brugia malayi*. Mol Biochem Parasitol. 1987;23(3):275–84. Epub 1987/04/01. . 3110617 10.1016/0166-6851(87)90034-x

[60] Talari ACS, Movasaghi Z, Rehman S, Rehman IU. Raman spectroscopy of biological tissues. Appl Spect Reviews. 2015;50(1):46–111. 10.1080/05704928.2014.923902

[61] Matthaus C, Krafft C, Dietzek B, Brehm BR, Lorkowski S, Popp J. Noninvasive imaging of intracellular lipid metabolism in macrophages by raman microscopy in combination with stable isotopic labeling. Anal Chemistry. 2012;84:8549–56. 10.1021/ac3012347 22954250

[62] Naemat A, Elsheikha HM, Al-Sandaqchi A, Kong K, Ghita A, Notingher I. Analysis of interaction between the apicomplexan protozoan *Toxoplasma gondii* and host cells using label-free Raman spectroscopy. The Analyst. 2015;140(3):756–64. Epub 2014/11/26. 10.1039/c4an01810a . 25422831

[63] Kubata BK, Kabututu Z, Nozaki T, Munday CJ, Fukuzumi S, Ohkubo K, et al A key role for old yellow enzyme in the metabolism of drugs by *Trypanosoma cruzi*. J Exp Med. 2002;196(9):1241–51. Epub 2002/11/06. 12417633 10.1084/jem.20020885 PMC2194105

