# Supplementary figures and images for: Lipid Body Organelles within the Parasite Trypanosoma cruzi: A Role for Intracellular Arachidonic Acid Metabolism
Source: PLoS One. 2016 Aug 4;11(8):e0160433. doi: 10.1371/journal.pone.0160433 (PMC4973985; doi:10.1371/journal.pone.0160433)

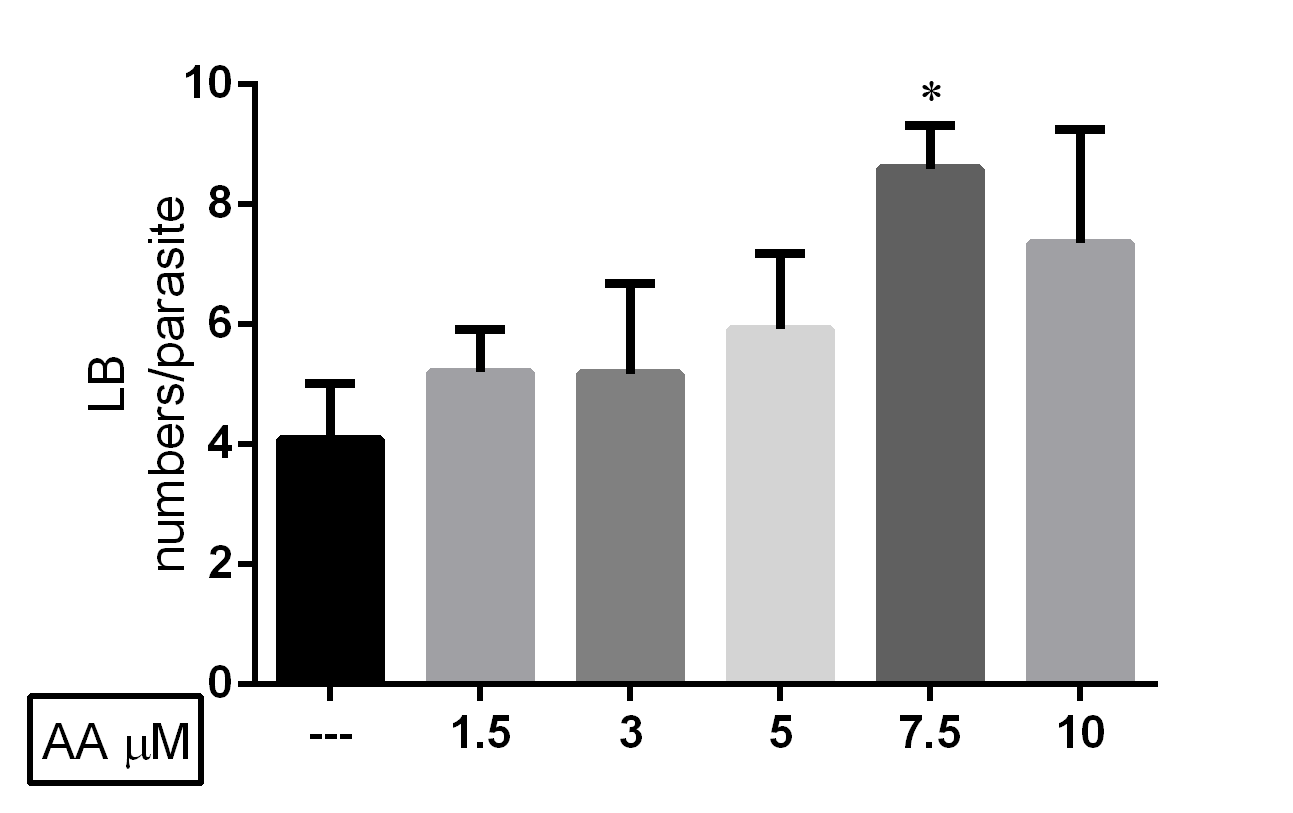

Supplement: S2 Fig — The dose-response curve of LB genesis was analyzed 1 h after stimulation with AA (1–10 μm). LBs were visualized and enumerated using osmium staining. Results were expressed as mean ± SEM, from at least 3 experiments. (TIF) [file pone.0160433.s002.tif]

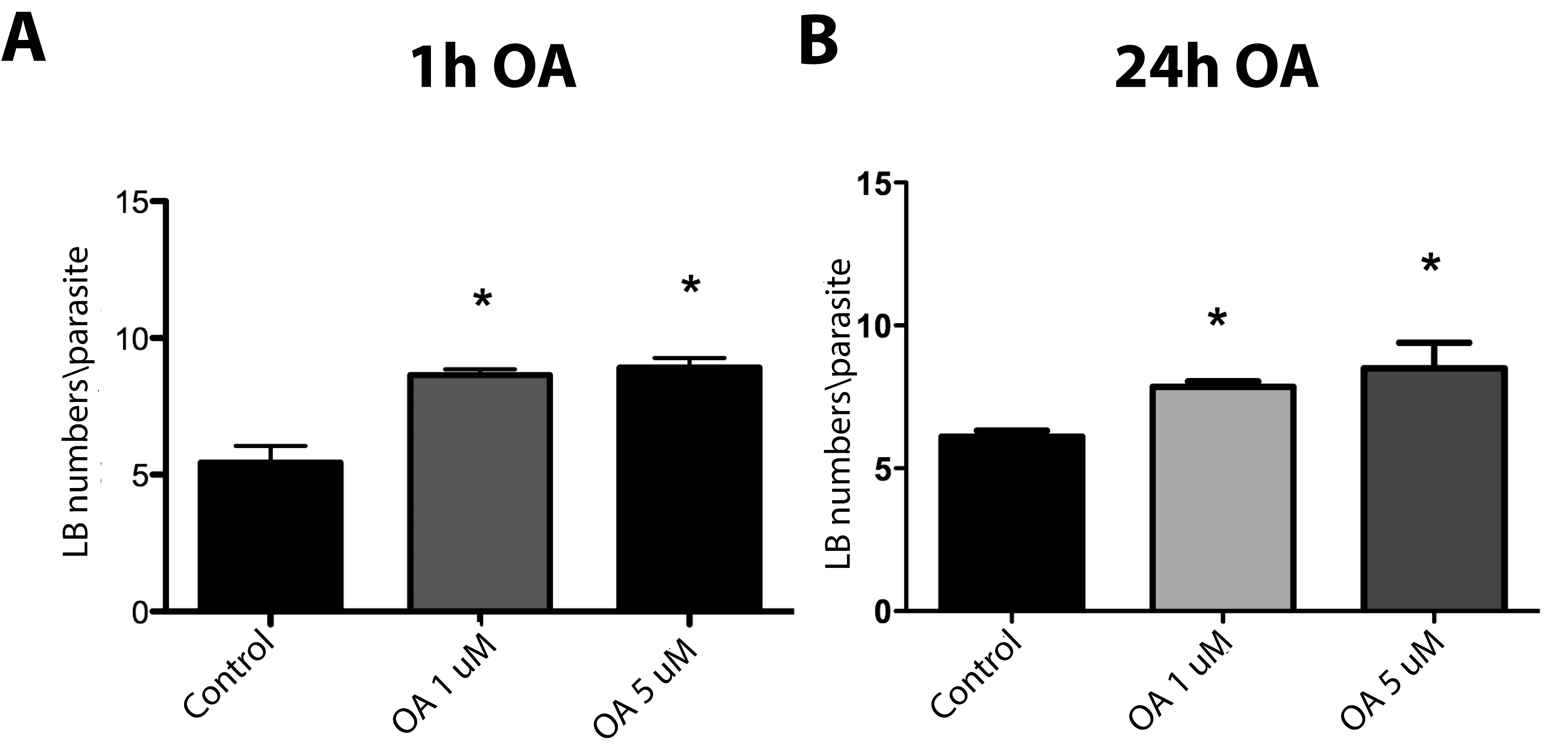

Supplement: S4 Fig — Bars represent the mean ± SEM of LBs per parasite from 50 consecutevely counted parasites from at least 4 independent experiments. * P < 0.05 between groups. Cells were enumerated using osmium staining. (TIF) [file pone.0160433.s004.tif]
